# Airway management and outcomes in surgical drainage of severe odontogenic infections: a retrospective cohort study

**DOI:** 10.1016/j.bjane.2026.844756

**Published:** 2026-04-24

**Authors:** Karam Azem, Adham Kashkush, Benjamin Zribi, Eitan Mangoubi, Shai Fein, Roussana Aranbitski, Sharon Orbach-Zinger, Esmat Najjar, Philip Heesen, Gal Avishai, Dror Bar Hai, Gavriel Chaushu, Daya Masri

**Affiliations:** a Tel Aviv University, Rabin Medical Centre and the Gray Faculty of Medical & Health Sciences, Beilinson Hospital, Department of Anesthesiology, Tel Aviv, Israel; b Ben-Gurion University of the Negev, Assuta Medical Centre and the Faculty of Health Sciences, Department of Anesthesiology, Beer-Sheva, Israel; c Technion-Israel Institute of Technology, Carmel Medical Centre, and the Ruth and Bruce Rappaport Faculty of Medicine, Department of Anesthesiology, Haifa, Israel; d Tel Aviv University, Rabin Medical Centre and the Gray Faculty of Medical & Health Sciences, Beilinson Hospital, Department of Otorhinolaryngology - Head & Neck Surgery, Tel Aviv, Israel; e University of Zurich, Epidemiology, Biostatistics and Prevention Institute and the Faculty of Medicine, Zurich, Switzerland; f Tel Aviv University, Rabin Medical Centre and the Gray Faculty of Medical & Health Sciences, Beilinson Hospital, Department of Oral and Maxillofacial Surgery, Tel Aviv, Israel

**Keywords:** Abscess, Airway Management, Focal Infection: Dental, Laryngoscopy, Sepsis, Trismus

## Abstract

**Background:**

Severe odontogenic infections requiring surgical drainage pose significant airway management challenges. This study aimed to compare perioperative characteristics and clinical outcomes between patients managed with conventional laryngoscopy versus awake fiberoptic intubation, to identify factors associated with the selection of airway management technique, and to determine predictors of hospital length of stay.

**Methods:**

This single-center retrospective study included 85 adult patients who underwent surgical drainage of severe odontogenic infections under general anesthesia between 2015 and 2024. The primary objective was to identify factors associated with awake fiberoptic intubation selection. Secondary objectives included comparison of perioperative outcomes and identification of predictors of hospital discharge. Multivariable logistic regression and Cox proportional hazards modeling were used.

**Results:**

Of 85 patients, 60% were managed with AFOI. Multivariable analysis identified trismus (OR = 6.67, 95% CI 1.53, 39.53, p = 0.010) and higher BMI (OR = 1.15, 95% CI 1.03, 1.33, p = 0.013) as independent predictors of AFOI selection. AFOI patients experienced more complex postoperative courses, with intraoperative tracheostomy, ICU admission, and septic shock occurring exclusively in this group. Spontaneous ventilation postoperatively was associated with earlier discharge (HR = 2.14, 95% CI 1.23, 3.73, p = 0.007), while septic shock (HR = 0.26, 95% CI 0.07, 0.91, p = 0.036) and lower BMI (HR = 0.95, 95% CI 0.90, 1.00, p = 0.040) were associated with delayed discharge.

**Conclusion:**

AFOI was preferentially selected for anatomically complex odontogenic infections based on objective criteria, particularly trismus and elevated BMI. The ability to achieve spontaneous ventilation postoperatively serves as a key prognostic indicator, while septic shock is strongly associated with prolonged recovery. These findings support evidence-based airway management protocols and early identification of high-risk patients to optimize outcomes in this challenging population.

## Introduction

Deep neck space infections of odontogenic origin, a severe manifestation of dental pathology, can rapidly disseminate through fascial planes, potentially resulting in life-threatening complications, including airway compromise, sepsis, and even death.^1^, ^2^, ^3^ Surgical drainage of these abscesses under general anesthesia introduces significant anesthetic challenges. Common presentations include trismus, floor-of-mouth edema, pharyngeal swelling, and distorted airway anatomy, all of which significantly complicate airway management.^4^^,^^5^

Several airway management strategies have been described for this patient population, ranging from conventional direct laryngoscopy to video laryngoscopy, Awake Fiberoptic Intubation (AFOI), and tracheostomy under local anesthesia.^6^^,^^7^ However, the optimal approach remains unresolved, with each technique carrying unique benefits and risks in the setting of distorted anatomy and potentially compromised airways.

Current international guidelines distinguish between approaches for anticipated and unanticipated difficult airways, providing structured algorithms to guide decision-making.^8^^,^^9^ For patients with severe odontogenic infections, where a difficult airway is often anticipated, these guidelines suggest considering awake techniques or securing the airway before induction when appropriate. However, the final approach must be tailored to each patient's clinical presentation.

The incidence of severe odontogenic infections requiring hospital admission has been increasing in developed countries,^10^ with these infections remaining clinically challenging despite modern treatment.^11^ The multifaceted nature of these infections demands a comprehensive understanding of how patient characteristics, anatomical spread patterns, and airway management decisions interact to influence clinical outcomes.^12^^,^^13^

While existing literature provides insights into surgical management of these infections, critical knowledge gaps persist regarding the factors that guide airway management decisions and their downstream effects on patient outcomes and hospital resource utilization. In this descriptive, exploratory study, we present a single-center experience aimed at describing the perioperative characteristics and outcomes of patients managed with different airway techniques, identifying factors influencing the choice of airway management, and determining predictors of hospital length of stay, rather than evaluating the causal impact of airway management strategy on clinical outcomes.

## Methods

### Ethics, design, and settings

This retrospective cohort study was conducted at Rabin Medical Center, Beilinson Hospital, Israel. Ethical approval (0202-22-RMC) was provided by the Institutional Review Board (Chairperson Prof. Ran Tur-Kaspa). This manuscript adheres to the STROBE statement (Supplementary Table S3).^14^

### Study population

All adult patients (≥ 18 years) who underwent surgical drainage of severe odontogenic infections under general anesthesia between January 2015 and December 2024 were consecutively included. Exclusion criteria included arrival at the operating room already intubated and mechanically ventilated, awake tracheostomy before anesthesia induction, and substantially incomplete anesthesia or surgical documentation.

### Anesthetic and surgical care

All patients underwent comprehensive preoperative anesthesia evaluation, with particular emphasis on airway assessment, given the presence of severe odontogenic infection. Preoperative airway evaluation included assessment for trismus, floor-of-mouth involvement, pharyngeal edema, and overall infection spread pattern. Standard monitoring, according to the American Society of Anesthesiologists (ASA) guidelines, was implemented for all patients, including electrocardiography, non-invasive blood pressure, pulse oximetry, capnography, and temperature monitoring. Additional monitoring modalities were applied based on individual patient comorbidities and clinical presentation.

Patients were managed with one of two airway approaches: laryngoscopy after induction (laryngoscopy group) or AFOI before induction (AFOI group). The airway management strategy was determined by the attending anesthesiologist based on preoperative assessment and clinical judgment rather than a formal institutional protocol, with special consideration given to the extent and location of infection, presence of trismus, and anticipated surgical approach.

For patients in the laryngoscopy group, anesthesia was induced with intravenous agents, and neuromuscular blocking agents were administered to facilitate endotracheal intubation. The choice between direct laryngoscopy and video laryngoscopy was based on anesthesiologist preference and anticipated difficulty.

For patients in the AFOI group, topical anesthesia of the airway was achieved using lidocaine spray and/or nebulization. Conscious sedation was provided with various combinations of midazolam, fentanyl, ketamine, and/or propofol, carefully titrated to maintain spontaneous ventilation and patient cooperation while ensuring comfort. Nasotracheal intubation was preferred for most AFOI cases to optimize surgical access.

Following successful airway securing in both groups, anesthesia was maintained with inhalational agents (typically isoflurane or sevoflurane) with or without nitrous oxide, supplemented with opioid analgesics as needed. Fluid management, antibiotic administration, and additional medications were provided according to standard institutional protocols.

Maxillofacial surgeons performed surgical drainage following standard procedures for abscess treatment. Intraoperative tracheostomy was performed when deemed necessary based on the extent of infection and anticipated postoperative airway compromise. Decisions regarding postoperative airway management, including extubation versus continued mechanical ventilation, were made collaboratively by the anesthesia and surgical teams based on the extent of infection, degree of airway edema, and overall patient condition.

Postoperatively, patients were transferred to the Post-Anesthesia Care Unit (PACU) and monitored according to institutional protocols. Patients requiring continued airway support or hemodynamic monitoring were admitted to the Intensive Care Unit (ICU). All patients received appropriate antibiotic therapy, analgesics, and supportive care throughout their hospital stay.

### Study outcomes

The primary objective was to identify factors associated with the selection of AFOI versus conventional laryngoscopy. Secondary objectives were to compare perioperative characteristics and clinical outcomes between groups, and to determine predictors of hospital length of stay.

### Measurements and data collection

Data were extracted from electronic medical record systems by trained investigators with domain expertise: anesthesia and operative data were extracted from Metavision (iMDsoft, Israel) by two anesthesiologists, and clinical and surgical data from Chameleon™ (Elad Health, Israel) by two oral and maxillofacial surgeons. Data accuracy was verified through cross-checking between investigators within each domain. Demographic data included age, gender, Body Mass Index (BMI), ASA physical status, active smoking status, and relevant comorbidities (chronic obstructive pulmonary disease, diabetes mellitus, hypertension, ischemic heart disease, and psychological disorders).

Infection characteristics were documented, including etiology (peri-implant disease, pericoronitis, post-extraction infection, or pulp necrosis) and anatomical spaces involved (anterior, masticator, peri-mandibular, and pharyngeal spaces). The presence of trismus, defined as limited mouth opening (interincisal distance < 20 lt; 20 mm), was also recorded.

Preoperative data included, vital signs (heart rate, mean arterial pressure, and oxygen saturation) prior to anesthesia induction. Airway management details were recorded, including the technique used (direct laryngoscopy, video laryngoscopy, or AFOI), medications administered for induction or sedation (propofol, midazolam, ketamine, fentanyl), neuromuscular blocking agents (succinylcholine, rocuronium), endotracheal tube characteristics (size, route), and time required to secure the airway (defined as the time from patient entry into the operating room until confirmation of successful endotracheal intubation).

Postoperative data included ventilation status at the conclusion of surgery (spontaneous versus mechanical ventilation), duration of mechanical ventilation if applicable, PACU length of stay, occurrence of septic shock, ICU admission and length of stay, and total hospital length of stay.

### Statistical methods

Descriptive statistics were used to summarize the data. The distribution of continuous variables was assessed visually using histograms and Q-Q plots. Continuous variables were presented as median (25^th^, 75^th^ percentiles). Hodges-Lehmann estimators with 95% Confidence Intervals (95% CI) were calculated to quantify median differences between groups for continuous variables. Risk differences with 95% CI were calculated for categorical variables. Comparisons between groups were performed using the Mann-Whitney *U*-test for continuous variables and Fisher's exact test for categorical variables. Categorical variables were presented as counts and percentages (%). Given the limited Events Per Variable (EPV < 10), firth penalized logistic regression was used for all logistic analyses to address potential sparse data bias. Univariate logistic and Cox regression analyses were performed to screen potential predictors. Variables with p < 0.20 in univariate analysis were included in the multivariable models. Multivariable logistic regression was performed to identify factors associated with AFOI selection. Hospital length of stay was analyzed using Kaplan-Meier survival analysis and Cox proportional hazards regression; the proportional hazards assumption was assessed using Schoenfeld residuals. Multicollinearity was assessed using Variance Inflation Factors (VIF). Results were presented as Odds Ratios (OR) with 95% CI for logistic regression and Hazard Ratios (HR) with 95% CI for Cox regression, representing the probability of hospital discharge at any time point. Patients with missing data were excluded from the respective analyses (complete case analysis). All statistical tests were two-sided; p < 0.05 was considered statistically significant. Statistical analyses were conducted using R statistical software (version 4.4.1).

## Results

**Figure 1:**
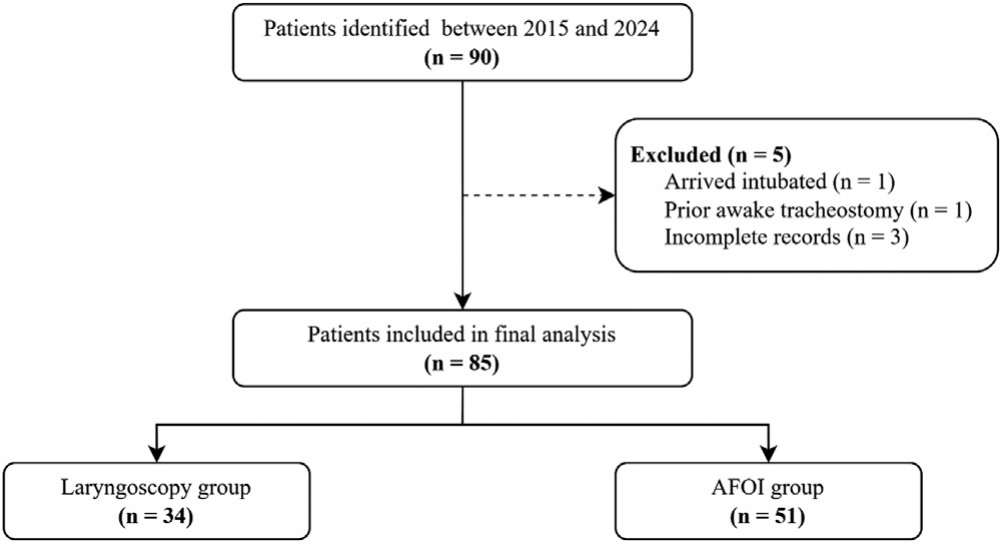
Patient inclusion flow chart. AFOI, awake fiberoptic intubation.

A total of 90 consecutive patients who underwent surgical drainage of severe odontogenic infections under general anesthesia were identified between 2015 and 2024. After exclusions, 85 patients were included in the final analysis. The patient inclusion flow chart is presented in Figure 1. Patients were divided into two groups based on airway management technique: laryngoscopy (n = 34, 40.0%) and AFOI (n = 51, 60.0%).

The median age of the cohort was 39.0 years (26.0, 48.0), with similar distribution between groups. Males comprised 48.2% of the overall cohort. Most patients were classified as ASA I or II (93.0%) and nearly half were active smokers (47.1%). Pulp necrosis was the predominant etiology of infection (55.3%), followed by post-extraction infection (32.9%). Peri-mandibular spaces were most involved (77.6%), followed by pharyngeal spaces (47.1%). Patients with pharyngeal space involvement (58.8% vs. 29.4%, p = 0.009) and trismus (92.2% vs. 61.8%, p < 0.001) were more frequently managed with AFOI. Median BMI was significantly higher in the AFOI group (24.9 vs. 22.3 kg.m^-2^, p = 0.024). Detailed patient characteristics are presented in Table 1.

**Table 1:** Baseline characteristics and clinical variables stratified by airway management technique.

	Overall (n = 85)	Laryngoscopy (n = 34)	AFOI (n = 51)	Effect size (95% CI)	p-value
**Age, y**	39.0 (26.0, 48.0)	32.5 (26.0, 52.5)	40.0 (27.5, 46.5)	-0.0 (-6.0, 7.0)	0.971
**Male sex**	41 (48.2%)	13 (38.2%)	28 (54.9%)	16.7 (-4.6, 38.0)	0.184
**BMI, kg.m^-2^**	23.3 (21.1, 28.6)	22.3 (20.3, 26.1)	24.9 (22.0, 29.4)	2.4 (0.3, 4.5)	**0.024**
**ASA physical status**					0.196
Class I	27 (31.8%)	7 (20.6%)	20 (39.2%)	18.6 (-0.5, 37.7)	
Class II	52 (61.2%)	25 (73.5%)	27 (52.9%)	-20.6 (-40.8, -0.4)	
Class III	5 (5.9%)	2 (5.9%)	3 (5.9%)	0.0 (-10.2, 10.2)	
Class IV	1 (1.2%)	0 (0.0%)	1 (2.0%)	2.0 (-1.8, 5.8)	
**Active smoking**	40 (47.1%)	18 (52.9%)	22 (43.1%)	-9.8 (-31.4, 11.8)	0.387
**Background disease**					
COPD	4 (4.7%)	2 (5.9%)	2 (3.9%)	-2.0 (-11.5, 7.6)	1.000
Diabetes mellitus	10 (11.8%)	7 (20.6%)	3 (5.9%)	-14.7 (-29.8, 0.3)	0.081
Hypertension	8 (9.4%)	4 (11.8%)	4 (7.8%)	-3.9 (-17.0, 9.2)	0.708
Ischemic heart disease	2 (2.4%)	2 (5.9%)	0 (0.0%)	-5.9 (-13.8, 2.0)	0.157
Psychological disorder	6 (7.1%)	3 (8.8%)	3 (5.9%)	-2.9 (-14.5, 8.6)	0.679
**Infection etiology**					
Peri-implant disease	5 (5.9%)	3 (8.8%)	2 (3.9%)	-4.9 (-15.8, 6.0)	0.385
Pericoronitis	5 (5.9%)	2 (5.9%)	3 (5.9%)	0.0 (-10.2, 10.2)	1.000
Postextraction infection	28 (32.9%)	10 (29.4%)	18 (35.3%)	5.9 (-14.3, 26.0)	0.642
Pulp necrosis	47 (55.3%)	19 (55.9%)	28 (54.9%)	-1.0 (-22.5, 20.6)	1.000
**Anatomical spaces involved**					
Anterior	18 (21.2%)	10 (29.4%)	8 (15.7%)	-13.7 (-32.0, 4.6)	0.176
Masticator	17 (20.0%)	6 (17.6%)	11 (21.6%)	3.9 (-13.2, 21.0)	0.785
Peri-mandibular	66 (77.6%)	25 (73.5%)	41 (80.4%)	6.9 (-11.5, 25.3)	0.596
Pharyngeal	40 (47.1%)	10 (29.4%)	30 (58.8%)	29.4 (9.0, 49.8)	**0.009**
Trismus	68 (80.0%)	21 (61.8%)	47 (92.2%)	30.4 (12.5, 48.3)	**< 0.001**

Categorical variables are presented as counts (%). Continuous variables are presented as a median (25^th^, 75^th^ percentiles). Effect sizes presented as median difference (95% CI) for continuous variables and risk difference in percentage points (95% CI) for categorical variables; positive values indicate higher values in the AFOI group. The Mann-Whitney *U*-test for comparing medians, and Fisher's exact test is used for comparing proportions. p-values compare the laryngoscopy group with the AFOI group. AFOI, Awake Fiberoptic Intubation; ASA, American Society of Anesthesiologists; BMI, Body Mass Index; CI, Confidence Intervals; COPD, Chronic Obstructive Pulmonary Disease.

Preinduction vital signs showed a median heart rate of 90.0 bpm (80.0, 100.0), median mean arterial pressure of 100.0 mmHg (92.0, 108.0), and median oxygen saturation of 99.0% (98.0, 100.0).

Among laryngoscopy patients, direct laryngoscopy was used in 58.8% and video laryngoscopy in 41.2%. Anesthesia was induced with propofol (97.1%, median dose 150.0 mg [120.0, 200.0]), midazolam (50%, median dose 2.0 mg [1.0, 2.0]), ketamine (5.9%, median dose 22.5 mg [21.2, 23.8]), and fentanyl (88.2%, median dose 125.0 μg [100.0, 200.0]). Neuromuscular blockade was achieved with succinylcholine (41.2%, median dose 100.0 mg [81.2, 100.0]) or rocuronium (55.9%, median dose 50.0 mg [40.0, 50.0]).

For AFOI patients, sedation was provided with midazolam (80.4%, median dose 2.0 mg [1.0, 2.0]), ketamine (31.4%, median dose 32.5 mg [20.0, 50.0]), fentanyl (56.9%, median dose 100 μg [50.0, 100.0]), and propofol (29.4%, median dose 50.0 mg [40.0, 100.0]). Nasotracheal intubation was preferred for AFOI cases (94.1% vs. 41.2%, p < 0.001). Time to secure the airway was significantly longer with AFOI (21.0 [13.0, 28.0] vs. 12.0 [8.0, 21.5] minutes, p = 0.002).

In univariate analysis, factors associated with AFOI selection included male sex, higher BMI, diabetes mellitus, trismus, pharyngeal space involvement, and anterior space involvement (Supplementary Table 1). Multivariable logistic regression identified trismus (OR = 6.67, 95% CI 1.53, 39.53, p = 0.010) and higher BMI (OR = 1.15, 95% CI 1.03, 1.33, p = 0.013) as independent factors associated with AFOI selection (Table 2).

**Table 2:** Multivariable logistic regression analysis of factors associated with AFOI selection.

Variable	Adjusted OR (95% CI)	p*-*value
Male sex	1.30 (0.47, 3.59)	0.607
BMI, per kg.m^-2^	1.15 (1.03, 1.33)	**0.013**
Diabetes mellitus	0.76 (0.12, 4.71)	0.762
Trismus	6.67 (1.53, 39.53)	**0.010**
Pharyngeal space involvement	1.61 (0.51, 5.12)	0.413
Anterior space involvement	0.71 (0.21, 2.49)	0.586

Firth penalized logistic regression was used to address sparse data bias. The dependent variable is airway management technique (AFOI vs. laryngoscopy). Independent variables with p < 0.20 in univariate analysis were included in the model. Multicollinearity was assessed using VIF (all VIF ≤ 1.51). OR represents the increased likelihood of AFOI selection associated with each factor. AFOI, Awake Fiberoptic Intubation; BMI, Body Mass Index; CI, Confidence Interval; OR, Odds Ratio; VIF, Variance Inflation Factors.

Mechanical ventilation at surgery conclusion was more common in AFOI patients (52.9% vs. 29.4%, p = 0.045), as was prolonged PACU stay (median 6.0 [1.9, 32.1] vs. 2.3 [1.5, 4.5] hours, p = 0.037). Intraoperative tracheostomy was performed exclusively in the AFOI group (27.5% vs. 0.0%, p < 0.001). ICU admission occurred only among AFOI patients (17.6% vs. 0.0%, p = 0.010). Septic shock developed in 7.8% of AFOI patients. Detailed postoperative outcomes are presented in Table 3.

**Table 3:** Intraoperative details and postoperative outcomes.

	Overall (n = 85)	Laryngoscopy (n = 34)	AFOI (n = 51)	Effect size (95% CI)	p-value
**Intraoperative data**					
Endotracheal tube size, mm	7.0 (6.5, 7.0)	7.0 (6.6, 7.4)	6.5 (6.5, 7.0)	-0.5 (-0.5, -0.0)	**0.002**
Endotracheal tube route					
Nasal	62 (72.9%)	14 (41.2%)	48 (94.1%)	52.9 (35.2, 70.7)	**< 0.001**
Oral	23 (27.1%)	20 (58.8%)	3 (5.9%)	-52.9 (-70.7, -35.2)	**< 0.001**
Time to secure AW, min	17.0 (12.0, 25.0)	12.0 (8.0, 21.5)	21.0 (13.0, 28.0)	7.0 (2.0, 11.0)	**0.002**
Surgery time	33.0 (19.0, 42.0)	30.0 (19.0, 41.2)	33.0 (22.0, 43.5)	3.0 (-4.0, 10.0)	0.490
Intraoperative tracheostomy	14 (16.5%)	0 (0.0%)	14 (27.5%)	27.5 (15.2, 39.7)	**< 0.001**
**Postoperative data**					
Ventilation status at surgery conclusion					**0.045**
Spontaneous ventilation	48 (56.5%)	24 (70.6%)	24 (47.1%)	-23.5 (-44.1, -3.0)	
Mechanical ventilation	37 (43.5%)	10 (29.4%)	27 (52.9%)	23.5 (3.0, 44.1)	
Duration of mechanical ventilation (h)	28.0 (12.3, 45.6)	20.3 (8.9, 47.9)	30.1 (13.7, 42.1)	5.5 (-21.3, 32.9)	0.580
PACU length of stay	2.6 (1.7, 17.0)	2.3 (1.5, 4.5)	6.0 (1.9, 32.1)	1.2 (0.0, 9.9)	**0.037**
Septic shock	4 (4.7%)	0 (0.0%)	4 (7.8%)	7.8 (0.5, 15.2)	0.146
ICU admission	9 (10.6%)	0 (0.0%)	9 (17.6%)	17.6 (7.2, 28.1)	**0.010**
ICU length of stay, days	12.0 (9.8, 18.0)	-	12.0 (9.8, 18.0)		
Hospital length of stay	5.0 (3.0, 8.0)	4.0 (3.0, 6.8)	6.0 (3.0, 10.5)	1.0 (-0.0, 3.0)	0.059

Categorical variables are presented as counts (%). Continuous variables are presented as a median (25^th^, 75^th^ percentiles). Effect sizes presented as median difference (95% CI) for continuous variables and risk difference in percentage points (95% CI) for categorical variables; positive values indicate higher values in the AFOI group. The Mann-Whitney *U*-test for comparing medians, and Fisher's exact test is used for comparing proportions. The p-values compare the laryngoscopy group with the AFOI group. AFOI, Awake Fiberoptic Intubation; AW, Airway; ICU, Intensive Care Unit; PACU, Post-Anesthesia Care Unit.

**Figure 2:**
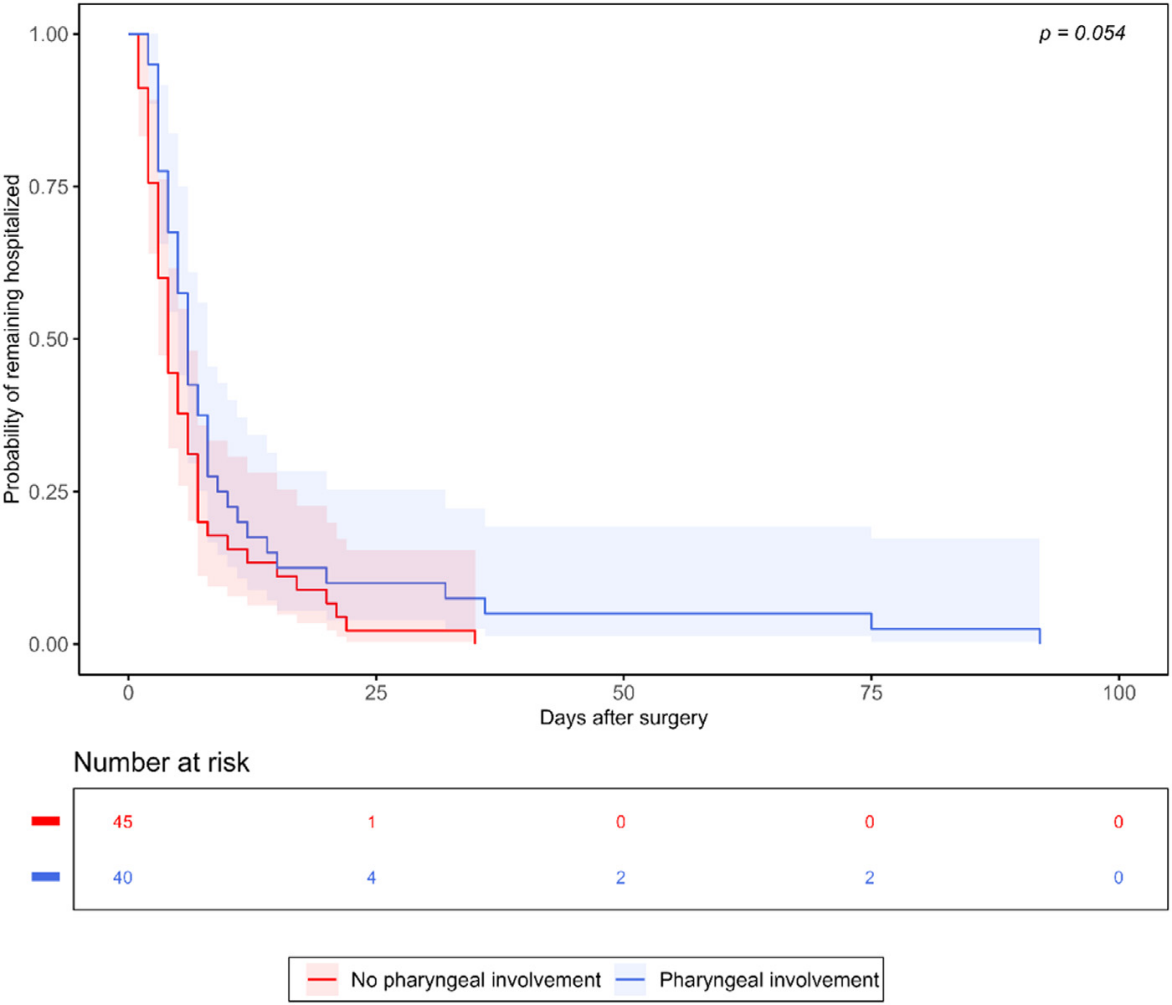
Kaplan-Meier analysis of hospital length of stay by pharyngeal space involvement. Kaplan-Meier curves showing time to hospital discharge stratified by pharyngeal space involvement. Patients without pharyngeal space involvement (red line) demonstrated shorter hospital stays compared to those with pharyngeal involvement (blue line). Log-rank test p = 0.054. Shaded areas represent 95% CI. Numbers at risk are shown below the plot. CI, Confidence Interval.

The overall median hospital length of stay was 5.0 days (3.0, 8.0). Univariate Cox regression analysis identified BMI, ASA class, AFOI technique, pharyngeal space involvement, peri-mandibular space involvement, spontaneous ventilation postoperatively, and septic shock as factors potentially associated with time to discharge (Supplementary Table 2). In the multivariable Cox proportional hazards model (Table 4), spontaneous ventilation postoperatively (HR = 2.14, 95% CI 1.23, 3.73, p = 0.007) was associated with earlier discharge, while septic shock (HR = 0.26, 95% CI 0.07, 0.91, p = 0.036) and lower BMI (HR = 0.95 per kg.m^-2^, 95% CI 0.90, 1.00, p = 0.040) were associated with delayed discharge. The relationship between pharyngeal space involvement and hospital length of stay is visualized in the Kaplan-Meier curve (Fig. 2, log-rank p = 0.054).

**Table 4:** Multivariable Cox regression analysis of factors associated with time to hospital discharge.

Variable	Adjusted HR (95% CI)	p*-*value
BMI, per kg.m^-2^	0.95 (0.90, 1.00)	**0.040**
ASA, per class	0.83 (0.55, 1.27)	0.396
AFOI (vs. laryngoscopy)	1.60 (0.91, 2.82)	0.102
Pharyngeal space involvement	0.63 (0.38, 1.04)	0.073
Peri-mandibular space involvement	0.75 (0.42, 1.34)	0.331
Spontaneous ventilation postoperatively	2.14 (1.23, 3.73)	**0.007**
Septic shock	0.26 (0.07, 0.91)	**0.036**

Analysis based on 79 patients with complete data. HRs represent the relative probability of hospital discharge at any time point. HR > 1 indicates faster discharge (shorter hospital stay). Variables with p < 0.20 in univariate analysis were included in the model. Multicollinearity was assessed using VIF (all VIF ≤ 1.51). The proportional hazards assumption was assessed using Schoenfeld residuals (global test p = 0.126). AFOI, Awake Fiberoptic Intubation; ASA, American Society of Anesthesiologists physical status; BMI, Body Mass Index; CI, Confidence Interval; HRs, Hazard Ratios; VIF, Variance Inflation Factors.

## Discussion

This retrospective cohort study of patients undergoing surgical drainage of severe odontogenic infections revealed several notable insights into airway management strategies and clinical outcomes. Our findings demonstrate that AFOI was selected for anatomically challenging cases, with selection primarily associated with trismus and elevated BMI. These patients experienced significantly different perioperative trajectories, likely reflecting underlying disease complexity rather than airway technique-related effects.

The striking association between trismus and AFOI selection aligns with emerging evidence establishing mouth opening as the most reliable predictor of airway difficulty in maxillofacial infections. Riekert et al. identified that trismus significantly predicted difficult airways and ICU admission.^15^ Our finding that 92.2% of AFOI patients presented with trismus, compared to 61.8% in the laryngoscopy group, reflects appropriate clinical decision-making based on this anatomical limitation. This is supported by difficult airway guidelines, which specifically recommend awake techniques when mouth opening is severely restricted.^8^^,^^9^ The longer time required to secure the airway with AFOI (21.0 vs. 12.0 minutes) reflects the technical demands of this approach rather than complications, as all patients were successfully intubated without reported adverse events.

BMI emerged as a unique bidirectional predictor: higher BMI was associated with AFOI selection (OR = 1.15 per kg.m^-2^, p = 0.013), while lower BMI was associated with delayed hospital discharge (HR = 0.95, p = 0.040). This dual association reflects the complex interplay between body habitus and infection severity. The association with AFOI selection aligns with established literature documenting increased airway management challenges in obese patients.^16^ However, the relationship between lower BMI and prolonged hospitalization may reflect underlying nutritional compromise or systemic illness affecting recovery capacity. Malnutrition has been independently associated with nosocomial infections and prolonged hospital stay.^17^, ^18^, ^19^

Pharyngeal space involvement emerged as a notable anatomical consideration, occurring in 58.8% of AFOI patients compared to 29.4% in the laryngoscopy group (p = 0.009). While not reaching statistical significance in multivariable analysis for AFOI selection, the trend toward prolonged hospitalization (HR = 0.63, p = 0.073) represents a hypothesis-generating finding that warrants further investigation in larger cohorts. This finding is consistent with existing literature identifying parapharyngeal and retropharyngeal space involvement as independent predictors of airway compromise and severe complications.^20^^,^^21^ The exclusive need for intraoperative tracheostomy in the AFOI group underscores the anatomical challenges and severity of infection involving critical airway structures, often necessitating temporary airway bypass.^22^^,^^23^

The ability to achieve spontaneous ventilation at surgery conclusion emerged as the strongest predictor of favorable outcomes (HR = 2.14 for earlier discharge, p = 0.007). This marker likely represents a composite indicator of infection severity, successful source control, and preserved physiologic reserve. The higher rate of postoperative mechanical ventilation in AFOI patients (52.9% vs. 29.4%, p = 0.045) reflects the underlying complexity of these cases rather than a consequence of the airway technique itself.

The exclusive occurrence of septic shock in the AFOI group, while based on only four cases, aligns with Weise et al.'s findings that 3.3% of hospitalized odontogenic infection patients develop septic complications requiring intensive support.^11^ All septic patients in their series required tracheostomy and prolonged ICU care, with 18.8% developing multiorgan dysfunction. Our finding that septic shock was strongly associated with delayed discharge (HR = 0.26, p = 0.036) emphasizes the need for early recognition and aggressive management of systemic complications. These observations are particularly relevant given recent epidemiological trends indicating rising ICU admission rates and hospital lengths of stay for odontogenic infections.^24^^,^^25^ In our study, 10.6% of patients required ICU admission, exclusively in the AFOI group, highlighting both the increasing severity of disease and the appropriate selection of advanced airway techniques for higher-risk patients.

Dexmedetomidine has emerged as a promising sedation agent for AFOI due to its ability to provide cooperative sedation with preserved spontaneous ventilation.^26^^,^^27^ However, its associated bradycardia and hypotension^28^ warrant caution in patients with sepsis or hemodynamic instability, as observed in our cohort.

Our findings support an individualized approach to airway management in severe odontogenic infections, with particular attention to trismus and elevated BMI. The prognostic significance of postoperative ventilation status and septic shock development aids patient counseling, resource allocation, and early identification of cases requiring intensive care. The observed pattern of AFOI selection for more complex cases, followed by appropriate postoperative management, reflects sound clinical judgment in perioperative care. From a practical standpoint, these findings support the use of trismus and elevated BMI as objective criteria for AFOI selection, and highlight the importance of postoperative ventilation planning and early sepsis recognition in institutional protocols for managing severe odontogenic infections.

### Limitations and future directions

This study has several limitations that warrant consideration. First, the retrospective single-center nature may introduce selection bias and limit generalizability to other institutions or healthcare settings, particularly as the extended study period (2015 to 2024) may encompass changes in clinical protocols or practice patterns, and availability of resources such as video laryngoscopy and dedicated maxillofacial surgical coverage. Second, the non-randomized selection of airway techniques creates potential confounding, as patients with more severe presentations are typically more likely to undergo AFOI. This selection bias, while clinically appropriate, prevents definitive conclusions about the superiority of either technique. Third, our modest sample size limits statistical power and the ability to perform detailed subgroup analyses. As severe odontogenic infections requiring surgical drainage under general anesthesia are relatively uncommon, we conducted a complete enumeration of all eligible cases during the study period rather than employing sample size calculations. This approach, while ensuring capture of all available data, limits statistical power for some comparisons. Fourth, reliance on medical records for clinical features, such as trismus, rather than standardized measurements, may introduce variability in assessment. Fifth, we lack long-term follow-up data beyond hospital discharge, which prevents an assessment of delayed complications or readmission rates.

Future research should include prospective studies with standardized assessment protocols and larger cohorts to better elucidate relationships between patient characteristics, infection patterns, airway management, and outcomes. The observed bidirectional association between BMI and outcomes warrants prospective validation with standardized nutritional assessments. Developing risk stratification tools incorporating predictors of prolonged hospitalization and ICU admission could optimize resource allocation. Investigating targeted interventions for high-risk patients, particularly those with pharyngeal space involvement or risk factors for septic shock, may improve outcomes in this vulnerable population.

## Conclusion

This retrospective analysis demonstrates that AFOI was preferentially selected for anatomically challenging cases of severe odontogenic infections, particularly those with trismus and elevated BMI. While these patients experience more complex perioperative courses, including higher rates of postoperative mechanical ventilation and ICU admission, these outcomes reflect underlying disease severity rather than technique-related complications. The ability to achieve spontaneous ventilation postoperatively emerges as a key prognostic indicator, while septic shock, though rare, is strongly associated with prolonged recovery. These findings support individualized airway management strategies based on objective clinical criteria and highlight the need for institutional preparedness to manage these increasingly complex infections.

## Data availability statement

Data may be obtained from the authors upon reasonable request, with the requisite permission from the Institutional Review Board of Rabin Medical Centre ‒ Beilinson Hospital.

## Funding

None.

## Conflicts of interest

The authors declare no conflicts of interest.

## Appendix Supplementary materials

Image, application 1

## Footnotes

Supplementary material associated with this article can be found in the online version at doi:10.1016/j.bjane.2026.844756.
