# Supplementary material for: Airway management and outcomes in surgical drainage of severe odontogenic infections: a retrospective cohort study
Source: Braz J Anesthesiol. 2026 Apr 24;76(3):844756. doi: 10.1016/j.bjane.2026.844756 (PMC13202563; doi:10.1016/j.bjane.2026.844756)
Supplement: Supplementary file 1 [file mmc1.docx]

**BJAN-D-25-00704_Supplementary Material**

**Supplementary Table 1** Univariate logistic regression analysis of factors associated with AFOI selection.

| **Variable** | **OR (95% CI)** | **p*-*value** |
| --- | --- | --- |
| Age, per year | 1.00 (0.97, 1.02) | 0.827 |
| Male sex | 1.93 (0.81, 4.70) | 0.136 |
| BMI, per kg.m^-2^ | 1.12 (1.01, 1.24) | **0.022** |
| ASA, per class | 0.68 (0.33, 1.37) | 0.283 |
| Active smoking | 0.68 (0.28, 1.61) | 0.380 |
| Hypertension | 0.64 (0.15, 2.66) | 0.531 |
| Diabetes mellitus | 0.26 (0.06, 0.97) | **0.045** |
| Trismus | 6.63 (2.16, 24.03) | **< 0.001** |
| Pharyngeal space involvement | 3.31 (1.36, 8.48) | **0.008** |
| Masticator space involvement | 1.25 (0.43, 3.83) | 0.688 |
| Peri-mandibular space involvement | 1.47 (0.53, 4.06) | 0.452 |
| Anterior space involvement | 0.46 (0.16, 1.27) | 0.134 |
| Infection etiology: Pulp necrosis | 0.96 (0.40, 2.28) | 0.934 |
| Infection etiology: Post-extraction infection | 1.29 (0.52, 3.30) | 0.587 |

Firth penalized logistic regression was used for all analyses to address sparse data bias. OR > 1 indicates increased likelihood of AFOI selection. Variables with p < 0.20 were included in the multivariable model (Table 2). AFOI, Awake Fiberoptic Intubation; ASA, American Society of Anesthesiologists physical status; BMI, Body Mass Index; CI, Confidence Interval; OR, Odds Ratio.

**Supplementary Table 2** Univariate Cox regression analysis of factors associated with time to hospital discharge.

| **Variable** | **HR (95% CI)** | **p-value** |
| --- | --- | --- |
| Age, per year | 0.99 (0.98, 1.01) | 0.210 |
| Male sex | 0.83 (0.54, 1.28) | 0.399 |
| BMI, per kg.m^-2^ | 0.95 (0.91, 1.00) | **0.034** |
| ASA, per class | 0.75 (0.54, 1.06) | 0.101 |
| AFOI (vs laryngoscopy) | 0.71 (0.46, 1.10) | 0.128 |
| Trismus | 1.06 (0.62, 1.83) | 0.829 |
| Pharyngeal space involvement | 0.66 (0.43, 1.02) | 0.061 |
| Masticator space involvement | 0.91 (0.53, 1.55) | 0.725 |
| Peri-mandibular space involvement | 0.66 (0.39, 1.10) | 0.112 |
| Spontaneous ventilation postoperatively | 2.77 (1.73, 4.45) | **< 0.001** |
| Septic shock | 0.26 (0.09, 0.75) | **0.012** |
| Infection etiology: Pulp necrosis | 1.20 (0.78, 1.84) | 0.417 |
| Infection etiology: Post-extraction infection | 0.85 (0.54, 1.35) | 0.498 |

HR > 1 indicates faster discharge (shorter hospital stay). Variables with p < 0.20 were included in the multivariable model (Table 4). Six patients with missing BMI data were excluded from the analyses. AFOI, Awake Fiberoptic Intubation; ASA, American Society of Anesthesiologists physical status; BMI, Body Mass Index; CI, Confidence Interval; HR, Hazard Ratio.

**Supplementary Table 3** STROBE Statement.

|  | | Item Nº | | Recommendation | Page Nº |  |
| --- | --- | --- | --- | --- | --- | --- |
| **Title and abstract** | | 1 | | (a) Indicate the study’s design with a commonly used term in the title or the abstract | 1 |  |
|  |  |  |  | (b) Provide in the abstract an informative and balanced summary of what was done and what was found | 2 |  |
| Introduction | | | | | |  |
| Background/Rationale | | 2 | | Explain the scientific background and rationale for the investigation being reported | 3 |  |
| Objectives | | 3 | | State specific objectives, including any prespecified hypotheses | 3 |  |
| Methods | | | | | |  |
| Study design | | 4 | | Present key elements of study design early in the paper | 4 |  |
| Setting | | 5 | | Describe the setting, locations, and relevant dates, including periods of recruitment, exposure, follow-up, and data collection | 4 |  |
| Participants | | 6 | | (a) *Cohort study* ‒ Give the eligibility criteria, and the sources and methods of selection of participants. Describe methods of follow-up; *Case-control study* ‒ Give the eligibility criteria, and the sources and methods of case ascertainment and control selection. Give the rationale for the choice of cases and controls; *Cross-sectional study* ‒ Give the eligibility criteria, and the sources and methods of selection of participants | 4 |  |
|  |  |  |  | (b) *Cohort study* ‒ For matched studies, give matching criteria and number of exposed and unexposed; *Case-control study* ‒ For matched studies, give matching criteria and the number of controls per case | Na |  |
| Variables | | 7 | | Clearly define all outcomes, exposures, predictors, potential confounders, and effect modifiers. Give diagnostic criteria, if applicable | 5, 6 |  |
| Data sources/ Measurement | | 8* | | For each variable of interest, give sources of data and details of methods of assessment (measurement). Describe comparability of assessment methods if there is more than one group | *5, 6* |  |
| Bias | | 9 | | Describe any efforts to address potential sources of bias | 6 |  |
| Study size | | 10 | | Explain how the study size was arrived at | Na |  |
| Quantitative variables | 11 | | Explain how quantitative variables were handled in the analyses. If applicable, describe which groupings were chosen and why | | 6 | |
| Statistical methods | 12 | | (a) Describe all statistical methods, including those used to control for confounding | | 6 | |
|  |  |  | (b) Describe any methods used to examine subgroups and interactions | | 6 | |
|  |  |  | (c) Explain how missing data were addressed | | 6 | |
|  |  |  | (d) *Cohort study* ‒ If applicable, explain how loss to follow-up was addressed; *Case-control study* ‒ If applicable, explain how matching of cases and controls was addressed; *Cross-sectional study* ‒ If applicable, describe analytical methods taking account of sampling strategy | | Na | |
|  |  |  | (e) Describe any sensitivity analyses | | Na | |
| Participants | 13* | | (a) Report numbers of individuals at each stage of study ‒ e.g., numbers potentially eligible, examined for eligibility, confirmed eligible, included in the study, completing follow-up, and analyzed | | 7 | |
|  |  |  | (b) Give reasons for non-participation at each stage | | 7 | |
|  |  |  | (c) Consider use of a flow diagram | | Na | |
| Descriptive data | 14* | | (a) Give characteristics of study participants (e.g., demographic, clinical, social) and information on exposures and potential confounders | | 7 | |
|  |  |  | (b) Indicate number of participants with missing data for each variable of interest | | 7 | |
|  |  |  | (c) *Cohort study* ‒ Summarise follow-up time (e.g., average and total amount) | | 7 | |
| Outcome data | 15* | | *Cohort study* ‒ Report numbers of outcome events or summary measures over time | | *7* | |
|  |  |  | *Case-control study ‒* Report numbers in each exposure category, or summary measures of exposure | | *7* | |
|  |  |  | *Cross-sectional study ‒* Report numbers of outcome events or summary measures | | *7* | |
| Main results | 16 | | (a) Give unadjusted estimates and, if applicable, confounder-adjusted estimates and their precision (e.g., 95% Confidence Interval). Make clear which confounders were adjusted for and why they were included | | 7, 8 | |
|  |  |  | (b) Report category boundaries when continuous variables were categorized | | Na | |
|  |  |  | (c) If relevant, consider translating estimates of relative risk into absolute risk for a meaningful time period | | Na | |
| Other analyses | 17 | | Report other analyses done ‒ e.g., analyses of subgroups and interactions, and sensitivity analyses | | Na | |
| Key results | 18 | | Summarise key results with reference to study objectives | | 9 | |
| Limitations | 19 | | Discuss limitations of the study, taking into account sources of potential bias or imprecision. Discuss both direction and magnitude of any potential bias | | 10, 11 | |
| Interpretation | 20 | | Give a cautious overall interpretation of results considering objectives, limitations, multiplicity of analyses, results from similar studies, and other relevant evidence | | 11 | |
| Generalizability | 21 | | Discuss the generalisability (external validity) of the study results | | 11 | |
| **Other information** |  | |  | |  | |
| Funding | 22 | | Give the source of funding and the role of the funders for the present study and, if applicable, for the original study on which the present article is based | | 12 | |
